# Edible Safety Assessment of Genetically Modified Rice T1C-1 for Sprague Dawley Rats through Horizontal Gene Transfer, Allergenicity and Intestinal Microbiota

**DOI:** 10.1371/journal.pone.0163352

**Published:** 2016-10-05

**Authors:** Kai Zhao, Fangfang Ren, Fangting Han, Qiwen Liu, Guogan Wu, Yan Xu, Jian Zhang, Xiao Wu, Jinbin Wang, Peng Li, Wei Shi, Hong Zhu, Jianjun Lv, Xiao Zhao, Xueming Tang

**Affiliations:** 1 Biotechnology Research Institute, Shanghai Academy of Agricultural Sciences, Shanghai, People’s Republic of China; 2 Key Laboratory of Agricultural Genetics and Breeding, Shanghai Academy of Agricultural Sciences, Shanghai, People’s Republic of China; 3 College of Life and Environment Sciences, Shanghai Normal University, Shanghai, People’s Republic of China; 4 Alberta Innovates-Technology Futures, Vegreville, Alberta, Canada; 5 National Center for Safety Evaluation of Drugs, Beijing Economic-Technological Development Area, Beijing, People’s Republic of China; 6 Life Science College, Ningxia University, Yinchuan, Ningxia, People’s Republic of China; Universidade Federal de Pelotas, BRAZIL

## Abstract

In this study, assessment of the safety of transgenic rice T1C-1 expressing Cry1C was carried out by: (1) studying horizontal gene transfer (HGT) in Sprague Dawley rats fed transgenic rice for 90 d; (2) examining the effect of Cry1C protein in vitro on digestibility and allergenicity; and (3) studying the changes of intestinal microbiota in rats fed with transgenic rice T1C-1 in acute and subchronic toxicity tests. Sprague Dawley rats were fed a diet containing either 60% GM *Bacillus thuringiensis* (Bt) rice T1C-1 expressing Cry1C protein, the parental rice Minghui 63, or a basic diet for 90 d. The GM Bt rice T1C-1 showed no evidence of HGT between rats and transgenic rice. Sequence searching of the Cry1C protein showed no homology with known allergens or toxins. Cry1C protein was rapidly degraded *in vitro* with simulated gastric and intestinal fluids. The expressed Cry1C protein did not induce high levels of specific IgG and IgE antibodies in rats. The intestinal microbiota of rats fed T1C-1 was also analyzed in acute and subchronic toxicity tests by DGGE. Cluster analysis of DGGE profiles revealed significant individual differences in the rats' intestinal microbiota.

## Introduction

Rice (*Oryza sativa* L.) is one of the most important cereal crops and represents approximately 23% of all calories consumed worldwide [1]. Extensive cultivation of modern high yielding varieties of crops has resulted in a significant increase in the yield of most food crops, including rice. However, this has also augmented the development towards monocultures, which often favors drastic increases in numbers of the insects that feed on these crops. Despite extensive use of pesticides, an estimated 37% of crop production is lost due to pests and diseases, with at least 13% directly due to insects [2]. Moreover, long-term and frequent use of chemical insecticides has destroyed the balance of the ecosystem. Therefore, better and more sophisticated forms of crop protection are important to ensure a stable food supply to meet the demand of an ever-increasing global population.

During the past decade, genetic transformation has promoted a number of crop varieties expressing transgene(s) from related or unrelated taxa. *Bacillus thuringiensis* (Bt) corn was genetically modified by introducing the Bt gene to control insect pests [3]. Bt can produce large crystalline parasporal inclusions (Cry proteins) during sporulation. Among them, the Cry1C protein encoded by *cry1C* is highly toxic to about 35–40 insect species including rice stem borers, *Spodoptera exigua*, beet armyworm and lepidopteran pests such as diamond back moth (*Plutella xylostella*) [4–6]. The Cry1C toxin can also be combined with *Cry1A* and *Cry1Aa* genes to develop two-toxin Bt crops, which can enhance the toxicity of Cry1C against *S*. *exigua* and *Helicoverpa armigera* [7]. Cry1C toxin has high species-specific toxicity against certain insects and can be used in developing transgenic crops to control lepidopteran pests.

Insect-resistant Bt-transgenic crops were first grown commercially in 1996 [8], and since then the planting area of Bt crops has increased steadily. Corn, cotton, canola and potatoes, which were genetically engineered with a range of different *Cry* genes are commercially grown around the world. Transgenic rice expressing Bt-Cry protein is resistant to most lepidopteran insect pests. The cultivation of the transgenic Bt-Cry rice has the potential to significantly decrease yield losses, to reduce the use of broad-spectrum chemical insecticides, and furthermore to reduce the levels of mycotoxins because of reduced larval attacks [9].

Like any newly developing technology, there have been worries about the potential risks of the pest-resistant Bt rice. The commercialization of genetically modified (GM) crops has raised concerns worldwide on biosafety [10, 11]. Universal commercialization of GM organisms has caused regulations in some countries to be enacted in order to protect consumers’ rights [12]. For example, in North America, regulators demand data on food safety, nutritional composition and a wide variety of environmental considerations before commercializing any GM crop. The food safety evaluation of GM crops includes tests for toxicology, allergenicity, horizontal gene transfer (HGT), anti-nutritional factors and intestinal microbiota.

HGT refers to the exchange of genetic material between different individual organisms and between single cell organelles. Different organisms can be the same species but with individual genetic differences, and can also be organisms with no genetic relationship. HGT, as opposed to vertical gene transfer (parent to offspring), breaks the boundaries of kinship, and thus, gene flow may become more complex. The transfer of DNA from GM crops might affect human health and safety through food chain transfer or deposition. It is reported that transgenic DNA components were not detected in muscle tissue when pigs and chickens were fed with GM soybean meal and corn [13]. However, in another study, when pregnant mice were fed high doses of bacterial DNA, the DNA fragments were found in the mouse embryos and newborns [14, 15]. The contradictory results have attracted great attention. With the rapid development of GM plants, an investigation of possible HGT between transgenic plants and intestinal microorganisms and animal cells is required. Thus, evaluation of HGT of transgenic Bt-cry1C rice T1C-1 to rats and intestinal microbiota was carried out in this study.

There are highly specific protein sequences in Cry1C proteins, but the sequence in humans differs from that in other species. Thus, safety assessment to evaluate the allergenicity of the Cry1C protein in human food or animal feed is absolutely necessary before application of Cry1C in rice. Experiments conducted with Bt rice expressing Cry1Ab/Cry1Ac protein have been reported [16, 17], but it is not known whether the Cry1C protein in transgenic rice is safe for humans. The Cry1C protein did not cause adverse effects in ICR (Institute of Cancer Research) mice when administered by gavage at a high dosage of 5 g (Cry1C protein)/kg body weight–the protein was degraded rapidly *in vitro* with simulated gastric or intestinal fluids [18]. The intestinal microbiota of ICR mice fed with Cry1C protein in an acute oral toxicity test was analyzed by denaturing gradient gel electrophoresis (DGGE), and showed that the Cry1C protein was safe for mice [19], however, its allergenicity has not been clearly investigated.

Another primary ecological concern about Bt plants is their potential effects on non-target organisms, such as intestinal microbiota [20]. DGGE was employed in the present study to determine the genetic diversity of complex microbial populations [21].

In this paper, the safety of transgenic rice TC-1 expressing Cry1C was assessed by detection in Sprague Dawley rats using HGT, *in vitro* digestibility, allergenicity and intestinal microbiota testing.

## Results

### Safety assessment of HGT

#### *cry1C* DNA detection in rats’ tissues

**Fig 1:**
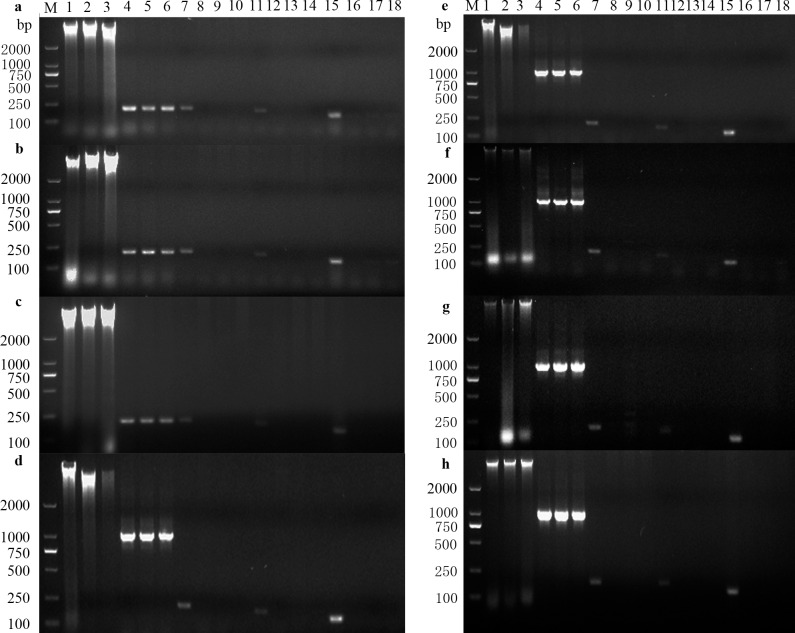
Safety assessment of HGT. PCR analysis of *cry1C* gene from masseter muscle (**a**), duodenum (**b**), ileum (**c**). M, DL2000 marker; 1–3, genomic DNA of T1C-1, Minghui 63 and control group, respectively; 4–6, *prl* of the genomic DNA of T1C-1, Minghui 63 and control group, respectively; 7, 35S promoter positive control; 8–10, 35S promoter of T1C-1, Minghui 63 and control group, respectively; 11, NOS terminator positive; 12–14, NOS terminator of T1C–1, Minghui 63 and control group, respectively; 15, exogenous gene control; 16–18, exogenous gene *cry1C* of T1C-1, Minghui 63 and control group, respectively. PCR analysis of *cry1C* gene from microbes in anaerobic cultures (**d**), *Salmonella* (**e**), *Lactobacilli* (**f**), *Streptococcus* (**g**), *E*. *coli* (**h**). M, DL2000 marker; 1–3, genomic DNA of T1C-1, Minghui 63 and control group, respectively; 4–6, bacterial 16S DNA of T1C-1, Minghui 63 and control group, respectively; 7, 35S promoter positive control; 8–10, 35S promoter of T1C-1, Minghui 63 and control group, respectively; 11, NOS terminator positive; 12–14, NOS terminator of T1C–1, Minghui 63 and control group, respectively; 15, exogenous gene control; 16–18, exogenous gene *cry1C* of T1C-1, Minghui 63 and control group, respectively.

The endogenous reference gene *prl* could be amplified from the genomic DNA of rat masseter muscle, duodenum and ileum tissue. The 35S promoter, NOS terminator and *cry1C* could not be amplified from rats fed with T1C-1, Minghui 63 rice and the control diet (Fig 1A–1C). It indicated that *cry1C* in GM rice T1C-1 did not transfer to rat tissues.

#### Testing of cry1C in intestinal microbiota

The endogenous reference gene *16s* was amplified from the genomic DNA of *E*. *coli*, *Salmonella*, *Lactobacilli* and *Streptococci* which were cultured from the intestinal feces of rats. The 35S promoter, NOS terminator and *cry1C* were successfully amplified in positive controls (Fig 1D–1H). However, 35S promoter, NOS terminator and *cry1C* could not be amplified from intestinal microbial DNA. This indicated that *cry1C* of T1C-1 did not transfer to the rat intestinal microbiota.

### Safety assessment of T1C-1 expressing Bt-Cry1C protein

#### Homology search with known toxins or allergens

The Cry1C sequence (GenBank AY955268.1) was analyzed in a database [22] (http://www.allergenonline.org/, version 9.0). It demonstrated no similarity between the Cry1C protein and any other known allergenic proteins in the database.

#### Characterization of the Cry1C protein derived from bacteria and rice

**Fig 2:**
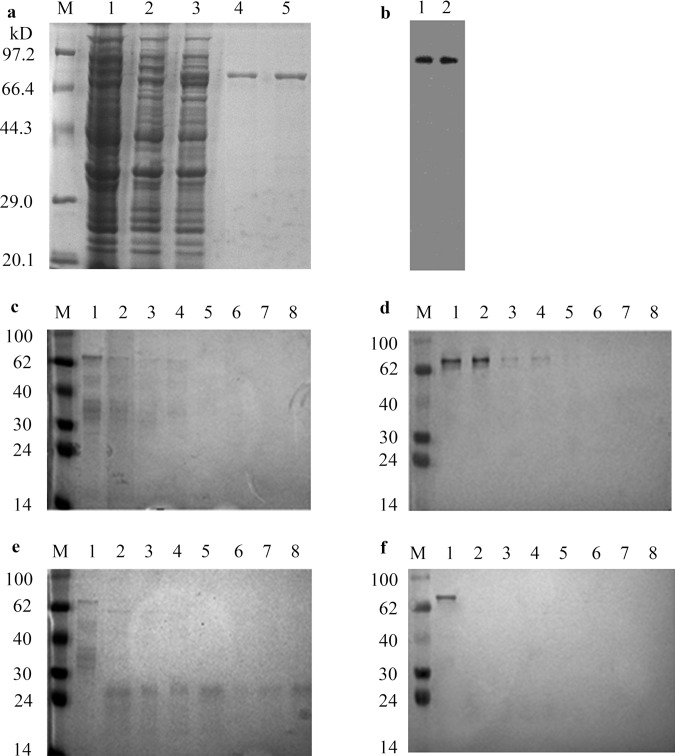
SDS-PAGE, western blot analysis of Cry1C protein and the protein degradation test in SGF and SIF. (**a**) M, molecular mass protein standards; 1–3, lysis supernatant of Transetta (DE3) cells, cells transformed with pET30a (+) and pET30a–Cry1C, respectively; 4, 5, Cry1C fusion protein. (**b**) Western blot analysis of Cry1C protein expressed in *E*. *coli* (lane 1) and the rice-derived Cry1C protein (lane 2). Gel staining (**c,e**) and western blot analysis (**d**,**f**) of fusion protein digested with simulated gastric fluid (SGF), simulated intestinal fluid (SIF), respectively. M, molecular mass protein standards; 1–8, 0 s, 15 s, 30 s, 60 s, 2 min, 10 min, 30 min and 60 min after digestion, respectively.

The Cry1C protein was expressed in *E*. *coli* DE3 (Fig 2A). It demonstrated that the recombinant Cry1C from *E*. *coli* could be an appropriate substitute for the protein isolated from the GM rice. Western blot showed that *E*. *coli*- and the GM rice-derived proteins had the same molecular weight and epitope accessibility (Fig 2B).

The recombinant Cry1C protein possessed the same insecticidal activity as the rice-derived Cry1C protein. With dosage of 25 μg of *E*. *coli*-derived Cry1C protein in 1 g of feed, mean mortality reached 95%; mean mortality in the T1C-1 rice group was 74.2% and in negative and positive controls reached 11.1 and 97.2%, respectively. These results suggested that the proteins of both origins had the same biochemical properties.

#### Degradation of the Cry1C protein in digestive fluids

The digestibility of the Cry1C protein was determined by using SGF (simulated gastric fluid) and SIF (simulated intestinal fluid) tests. The Cry1C protein was completely digested within 60 s in SGF (Fig 2C and 2D) and within 15 s in SIF (Fig 2E and 2F), while the reference protein were rapidly (15 s) and slowly (30 min) digested in the SGF/SIF assay, respectively.

#### Antigen-specific IgG response

The positive group of rats treated via ip injection with OVA (reference protein) showed significant immuno reactions; and their sera, which were diluted 20-fold, had a much higher level of specific-IgG antibody (0.390 ± 0.0290) than the negative group (0.111 ± 0.0078), with Positive/Negative (P/N) > 2. In contrast, the immune response of the Cry1C (0.169 ± 0.0163, 0.170 ± 0.0126) and adjuvant group (0.164 ± 0.0167) were basically equivalent to that of the negative group (P/N < 2). There were significant differences (P < 0.05) in values of IgG between the groups immunized with OVA and the adjuvant, and the groups immunized with OVA and the Cry1C protein. The results indicated that OVA triggered an obvious immunological reaction, but Cry1C protein did not.

#### Evaluation of IgE response

When fed allergic proteins, the immune system of animals could produce specific IgE antibodies, and ELISA was used to test their presence. The optical density (OD) values of sera for the OVA group (0.545 ± 0.0500) were significantly (P < 0.05) higher than those of the PBS group (0.152 ± 0.0102) (P/N > 2). In contrast, the OD values corresponding to the Cry1C protein (0.184 ± 0.0203) and adjuvant group (0.176 ± 0.0143) were similar to those of the negative group (P/N < 2). Thus the Cry1C protein did not evoke specific IgE antibodies.

### The change of intestinal microbiota in acute toxicity test

**Fig 3:**
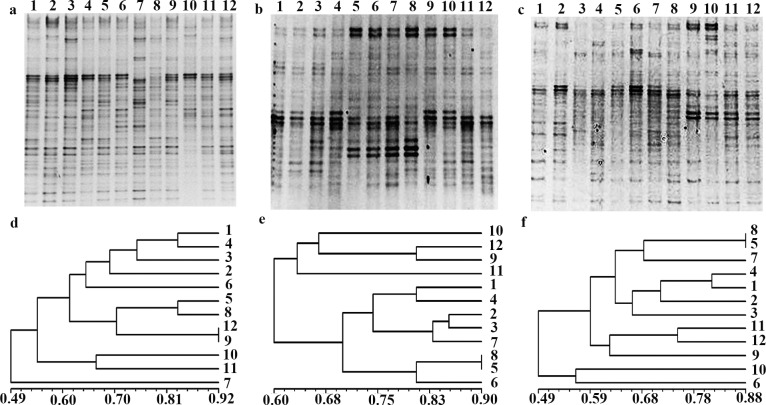
DGGE profiles and analysis by UPGAMA from rat fecal bacteria in the control and test group, respectively. (**a**) 1–4, 5–8, 9–12, samples from R1, R2 and R3 (the three rats in the control group) at 0, 1, 7 and 14 d after gavage respectively. (**b**) 1–4, 5–8, 9–12, samples from T1, T2 and T3 (the three rats in the test group) at 0, 1, 7 and 14 d, respectively. (**c**) 1–4, 5–8, 9–12, Samples from T4, T5, T6 (the other three rats in the test group) at 0, 1, 7 and 14 d, respectively. (**d–f**) analysis of rat fecal samples by UPGAMA after gavage.

The microorganism community was studied over a period of two weeks using DGGE analysis (Fig 3A–3C). Analysis of the nine samples showed stable microorganism profiles. The bands did not show apparent differences within the same rat fecal samples, indicating that composition of the microorganism community did not change over this period despite slight fluctuations in microorganism numbers. Only subject 2 of control samples and subjects 3 and 6 of test samples showed minor changes in their profiles, where a faint fragment appeared in the first and second sample but disappeared in the following samples.

Cluster analysis of DGGE profiles revealed that feeding rat with Cry1C protein for two weeks induced changes in the composition of fecal and cecal microbiota compared with the fecal profiles obtained before feeding (Fig 3D–3F).

### Subchronic toxicity test

#### Changes in intestinal microbiota

**Fig 4:**
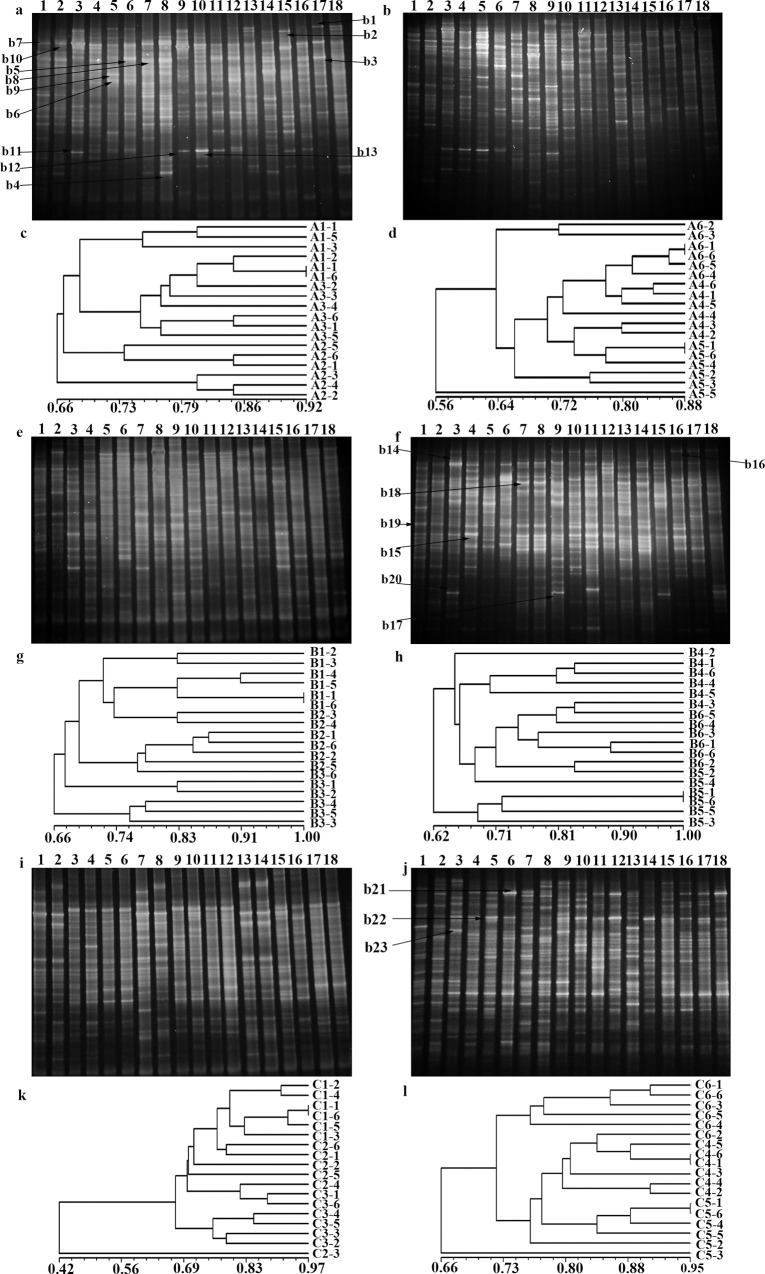
DGGE profiles and cluster analysis of the profiles from rat fecal bacteria. (**a**,**b**) DGGE profiles in test group fed daily with Bt rice T1C-1 expressing Cry1C. (**c**,**d**) Cluster analysis of DGGE profiles of a, b. (**e,f**) DGGE profiles in control group fed with rice Minghui 63. (**g,h**) Cluster analysis of DGGE profiles of g, h. (**i**,**j**) DGGE profiles in control group fed with untreated food. (**k,l**) Cluster analysis of DGGE profiles of i, j. **a**, 1–6, samples from test group A1; 7–12, samples from A2; 13–18, samples from A3 at 0, 30, 60, 80, 85 and 90 d after feeding with transgenic rice T1C-1 expressing Cry1C. **b**, 1–6, samples from A4; 7–11, samples from A5; 12–18, samples from A6 at 0, 30, 60, 80, 85 and 90 d after feeding with transgenic rice T1C-1 expressing Cry1C. **e**: 1–6: samples from control group B1; 7–12: samples from B2; 13–18: samples from B3 at 0, 30, 60, 80, 85 and 90 d after feeding with rice Minghui 63; **f**: 1–6: samples from B4; 7–12: samples from B5; 13–18: samples from B6 at 0, 30, 60, 80, 85 and 90 d after feeding with rice Minghui 63. **i**: 1–6: samples from control group fed untreated food daily C1; 7–12: samples from C2; 13–18: samples from C3 at 0, 30, 60, 80, 85 and 90 d after feeding with untreated food daily; **j**: 1–6: samples from C4; 7–12: samples from C5; 13–18: samples from C6 at 0, 30, 60, 80, 85 and 90 d after feeding with untreated food daily.

**Fig 5:**
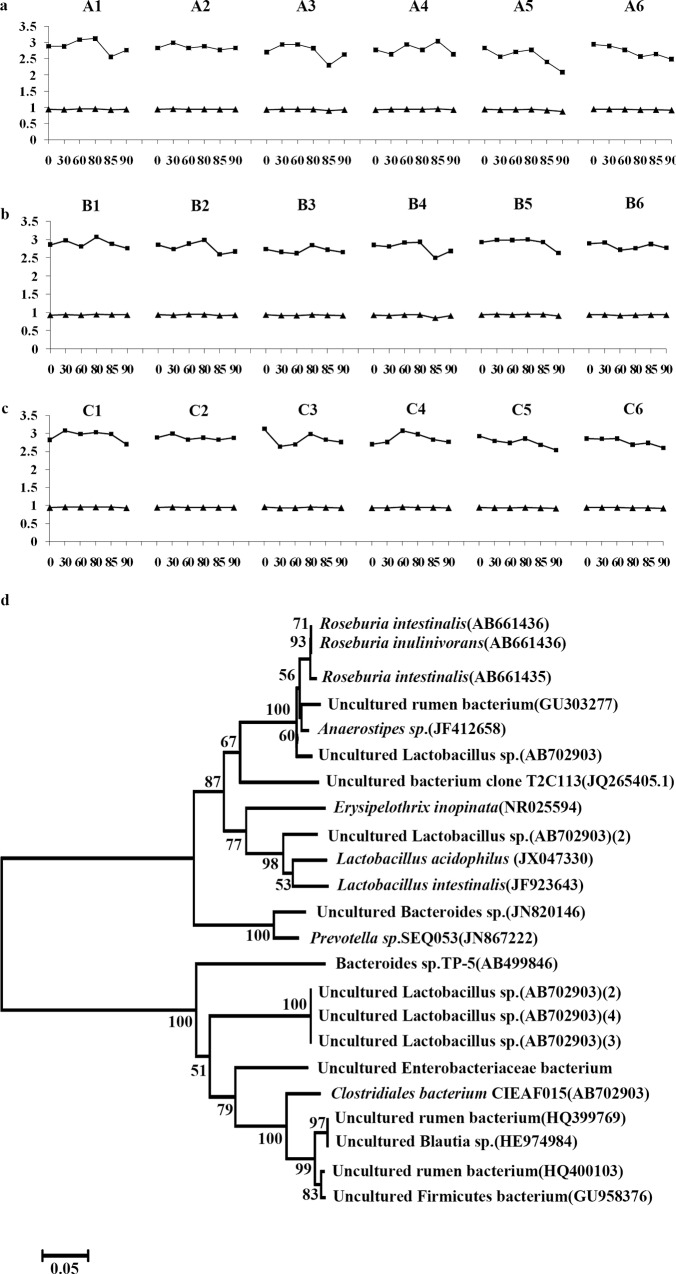
Shannon index (H) and Simpson’s index (D) of each band from DGGE bands and phylogenetic tree analysis of the 16S rDNA gene sequences of the obtained clones. **(a,b,c)** A1–A6, B1–B6 and C1–C6 have the same meanings as for Fig 4, ■The value of H, ▲ the value of D. (**d**) GenBank accession numbers are given in parentheses. The scale bar indicates the 0.05 evolutionary distance unit. Bootstrap values (percent) are shown at branch nodes.

PCR products of the V3 region of the 16S rDNA gene were analyzed by DGGE (Fig 4A, 4B, 4E, 4F, 4I and 4J). Representative bands were excised from DGGE gels and amplified before sequencing. The results of the identification are presented in S1 Table. The sequencing of DGGE bands showed that they possessed high similarity among the bands located relatively closely. *Lactobacillus acidophilus* was found in all groups [S1 Table (b3, b5, b14 and b23), Fig 4A, 4E and 4I]. The phylogenetic tree of 16S rDNA gene sequence of the obtained clones is presented in Fig 5.

#### Changes of the intestinal microbiota

DGGE analysis of fecal samples revealed changes of diverse microorganisms in test and control samples. The DGGE fingerprint patterns were highly reproducible in replicated samples. DGGE profiles of fecal samples showed diverse patterns, and bands of various intensities were identified (Fig 4A, 4B, 4E, 4F, 4I and 4J). Slight variation was observed among fecal samples in test and control groups at the same growth stage–with some common dominant bands, suggesting that dominant members of the bacterial community were ubiquitous within all fecal samples. This also suggested that Bt transgenic rice had little effect on the dominant microorganisms essential for long-term sustainability of rats. Cluster analysis revealed similar patterns for the bacterial community among the test group fed daily with Bt rice T1C-1 expressing Cry1C and the control group fed Minghui 63 (Fig 4C, 4D, 4G, 4H, 4K and 4L). DGGE banding patterns were divided into distinct clusters, with the highest of 92, 88, 100, 100, 97 and 95% similarity and the lowest of 66, 56, 66, 62, 42 and 62% similarity for bacterial communities, respectively. At the beginning stage, there were highly similar banding profiles for bacteria among samples from test and control groups. At the growth stages, there were various similarity degrees of bacteria among samples from test and control groups fed Minghui 63 and untreated food. Cluster analysis of DGGE patterns revealed that rat development had a stronger effect on intestinal microbiota than the Bt rice plant. The number of bands decreased as shown in Fig 5, there were no significant differences among samples from test groups fed with Bt rice T1C-1 expressing Cry1C, control groups fed with rice Minghui 63 and untreated food daily [F = 1.09, P > 0.05 (F_0.05_ = 3.68)]. Although the value of each rat varied from 0 to 60th day, the value at 90th day returned to the initial data of 0 d finally.

## Discussion

In this paper the safety of transgenic rice T1C-1 expressing Cry1C was assessed using three different approaches: (1) examining HGT between rats and transgenic rice; (2) studying the *in vitro* digestibility and allergenicity of transgenic rice T1C-1; and (3) studying the effects on intestinal bacteria in rats fed with transgenic rice T1C-1 in acute and subchronic toxicity tests.

PCR analysis showed no evidence of the exogenous *cry1C* gene in rat tissue samples. This indicated no HGT between the exogenous gene of transgenic rice and animal tissues. The exogenous gene showed no corresponding bands after amplification from various intestinal microbiota, suggesting no HGT between it and rat intestinal microbiota.

It has been reported that chloroplast DNA from plants in feed can be transferred to chicken muscle, liver, spleen and kidney tissues [23], but transgenic DNA from the feed composition was not detected in animal products such as muscle tissues and eggs [24–26]. As a single copy in the genome of transgenic crops, the exogenous gene has a lower detection probability after food is digested and broken down by digestive juices. Einspanier *et al*. reported that while an exogenous gene was detected in the duodenum of cattle fed a diet containing GM corn, the gene was not detected in fecal samples. Chamber *et al*. [27] found that although no exogenous gene fragments were detected in the small intestine and fecal samples of chickens fed a diet containing GM feed, the gene was detected in crop and stomach of chickens.

It is generally believed that the probability of HGT occurring in the digestive tract is small. HGT requires a variety of conditions occurring simultaneously and that can probably not be encountered along the digestive tract. The study in this paper supported this viewpoint and enriches the research data concerning HGT.

Numerous allergenicity studies have shown no significant adverse effects of the Cry proteins on body weight gain or clinical observations. Furthermore, no signs of pathogenicity to mammals, including humans, have been reported [28]. This demonstrates that the Cry1C protein has highly species-specific toxicity against certain insects and may be a sound candidate in controlling lepidopteran pests. For potential use of Cry1C, the protein was previously investigated by Cao [18], and now Cry1C with a different sequence as well as a different variety of rice was frequently used in study.

While new proteins produce and probably contact with humans, either by food or other way of exposure, identification of allergic potential is absolutely essential and important. In the Codex Alimentarius Commission, a standard framework for safety assessments of allergenicity was given in the Codex Alimentarius Commission [29]. For transgenes with no history of allergenicity, alternative approaches are required based on the structure and stability of the foreign protein. According to sequence search results, it demonstrated that the Cry1C protein used in this study had no homology with known toxins or allergens in AllergenOnline. Therefore, assessment by gastrointestinal digestion *in vitro* and by an animal model is necessary for its safety evaluation.

The Cry1C protein was digested in SGF and SIF within 60 and 15 s, respectively, suggesting that the protein may not show allergenic activity [30]. The Cry1C protein was hydrolyzed by digestive tract enzymes first, resulting in 90% activity loss before assayed by SDS-PAGE and western blot. Allergenicity will not produce if food allergens can not reach the intestinal mucosa. According to the results, the Cry1C protein was rapidly digested, and therfore its absorption was negligible.

The ip administration at 10 μg/ml OVA with adjuvant resulted in OVA-specific IgG and IgE responses in all rats. OVA-specific IgG and IgE were detected in the group immunized with OVA (P/N > 2). Anti-OVA IgG and IgE antibodies could not be detected in the group that was immunized with adjuvant (P/N < 2) and Cry1C protein (P/N < 2). There were remarkable differences in OD values of IgG and IgE between the group immunized with OVA and adjuvant, and the group immunized with OVA and Cry1C protein, suggesting that the majority of IgG and IgE in the group sensitized intraperitoneally with OVA was due to OVA. It was concluded that Sprague Dawley rats elicited serological responses (IgE antibody production).

The expressed Cry1C protein did not induce important levels of specific IgG and IgE antibodies in Sprague Dawley rats, suggesting that Cry1C protein is probably not an important allergenicity inducer. *In vitro* digestions of the recombinant Cry1C protein in SGF/SIF and the fact that the Cry1C protein did not induce significant levels of specific IgG and IgE antibodies in Sprague Dawley rats demonstrated that the Cry1C protein had low allergenicity.

The acute toxicity test showed that the Cry1C protein had no effect on Sprague Dawley rats. In the subchronic toxicity test, despite the apparent variations in microorganism communities at different growth stages, the transgenic rice T1C-1 expressing Cry1C protein had some effects on the microorganism communities of the gastrointestinal tract in rats at the first stage. However, when the feeding of rats with transgenic rice T1C-1 stopped at 80 d, the microorganism community began to recover and became stable at 90 d. The results are consistent with other studies concerning intestinal microbiota in mice fed with CrylC protein in an acute oral toxicity test [19,31], in which transgenic CrylC protein had no discernible effect on intestinal microbiota of mice in a two-week feeding experiment. In the present study, we adopted a 90-d dietary toxicity study of GM rice T1C-1. The results should provide valuable resources for future safety assessment of GM food crops.

*Lactobacillus acidophilus* belongs to the normal oropharyngeal and intestinal microbiota in both humans and animals. Throughout the experiment, *L*. *acidophilus* was present in all groups (S1 Table b3, b5, b14 and b23; Fig 4A, 4E and 4I). In the test group, the density of *L*. *acidophilus* changed after fed with transgenic rice T1C-1 expressing Cry1C, but was restored when fed with untreated food after 81 d. At the same time, there were similar changes in rats from the control groups. However, the density of *L*. *acidophilus* also changed with age. Bacteria from *Firmicutes* were also found in this study, and their changes were similar to those of *L*. *acidophilus*.

The H value is a measurement of DGGE band patterns, and provides a direct indication of the diversity of a microbial community–a high H value signifies high species diversity. Populations with more species and an even distribution of individual species show a greater diversity than populations with either a smaller number of species or a disproportionate abundance of individual species. There were no significant (P > 0.05) differences among samples from the test group fed Bt rice T1C-1 expressing Cry1C and the control groups fed either rice Minghui 63 or untreated food daily (Fig 5B).

Testing for 90 d provided no evidence for HGT between GM Bt rice T1C-1 and Sprague Dawley rats fed transgenic rice as 60% of their diet, in comparison with Minghui 63 and control groups. Sequence homology search of the Cry1C protein showed no homology with known allergens or toxins. Cry1C protein was rapidly degraded *in vitro* with simulated gastric and intestinal fluids. The expressed Cry1C protein did not induce high levels of specific IgG and IgE antibodies in rats, demonstrating that Cry1C was not likely a potential allergen. DGGE fingerprint patterns showed few differences in composition of bacterial communities in the intestinal microbiota among test groups fed transgenic rice T1C-1 and control groups fed Minghui 63 and untreated food daily, while the development course of rats had a stronger effect on intestinal microbial community compositions than transgenic rice T1C-1 did.

## Conclusions

In this study, we have assessed the edible safety of the GM rice T1C-1 with Cry1C for rats through evaluation of HGT, allergenicity and intestinal microbiota tests. The transgenic rice T1C-1 expressing Cry1C protein is likely safe for rats through the three evaluation tests. The findings provide insights into the debate on the safety of GM rice. The results reported in this paper offer new adequate envidences on edible assessment of GM rice, indicating that Bt rice T1C-1 expressing Cry1C protein is a safe new source of food.

## Methods

### Test materials

Bt rice (*O*. *sativa* L.) T1C-1 expressing Cry1C protein and its parent Minghui63 were obtained from Shanghai Agrobiological Gene Center. Seeds of T1C-1 and Minghui63 were produced in Hainan, China in 2011.

### Animals and housing

Sixty SPF Sprague Dawley rats (6–7 weeks old, 30 males and 30 females) were obtained from the Experimental Animal Center of Fudan University (Shanghai, China). All animals were kept in pairs in stainless steel wire cages at 22 ± 1°C and 40–60% relative humidity. Following one week of acclimatization, the rats were divided randomly into three groups, with 20 rats in each group (10 male and 10 female per treatment).

### Diet formulation and feeding

The purified or semi-synthetic rat diet was produced in-house based on the rodent diet AIN-93 [32]. The purified diet for the control group was based on corn starch without rice. Both test diets contained 60% ground rice flour, of either Minghui 63 or T1C-1 rice. Both diets were adjusted identically to assure an adequate supply of macronutrients and vitamins after substitution with 60% rice, but no adjustments were made to counterbalance differences in the constitution of the rice [7]. The rats were allowed free access to both food and water. Fresh fecal samples were collected on 0, 30, 60, 80, 85 and 90 d of the trial. Samples were stored on ice, and transferred to the lab within 30 min. The samples for DNA analysis were stored at –70°C.

### Animal dissection and enteric stool collection

Rat dissection was carried out in a bacteria-free operating environment. Two newly formed stool samples from the intestinal tract were taken from six rats (three males and three females) from each group. To determine aerobic and anaerobic bacteria, one sample was placed in 10 ml of sterilized saline media while the other into 10 ml of sterilized anaerobic media. The vials were stored at 4°C. For all rats in each group, the masseter muscle, ileum and duodenum were also sampled, washed in PBS, and stored at –80°C.

### Cultivation of rat intestinal microbiota

The aerobic and anaerobic bacterial samples were shaken vigorously on a vortex mixer for 2 min, followed by dilution with either 9 ml of normal saline or 1 ml of anaerobic media, respectively. For aerobic bacteria, a dilution gradient (10^−8^, 10^−9^ and 10^−10^) was adopted in solid culture. For anaerobic bacteria, 0.2 ml of culture of a dilution gradient (10^−8^, 10^−9^ and 10^−10^) was injected into the anaerobic tube containing 5 ml of MRS medium, and then coated evenly using the Hungate tube method. The cultivation conditions are presented in S2 Table.

#### DNA Extraction

The extraction of rat fecal samples was conducted according to TIANamp Stool Animal DNA Kit, and the genomic DNA was extracted according to TIANamp Genomic DNA Kit, Tiangen Biotech Co., Ltd (Beijing, China).

### The detection of HGT by PCR

Five pairs of specific primers were synthesized. Rat endogenous reference gene *prl*, *35s*, *nos* and *cry1C* gene were amplified from tissue DNA sample of each rat. The bacterial *prl*, *16s*, *nos* and *cry1C* gene were also amplified by PCR.

### Sequence similarity analysis of the Cry1C protein with known allergens or toxins

The sequence of Cry1C (GenBank AY955268.1) was aligned at the Protein Information Resource (PIR database http://pir.georgetown.edu/) using FASTA (version 2.4, 2002) [22]. There were 80 amino acid alignments used to search for known allergens with > 35% identity (http://www.allergenonline.org/, version 9.0). At least eight continuous amino acids are essential to result in IgE-dependent allergenicity [29], and eight-amino-acid matches were also searched. Proteins similar to Cry1C were identified manually with known allergens or toxic proteins.

### Protein production and characterization

The *cry1C* gene from GM rice T1C-1 was cloned and expressed in *E*. *coli* to obtain the Cry1C protein. The recombinant plasmid pET30a–Cry1C was transformed into *E*. *coli* DE3. The *E*. *coli* cells were harvested by centrifugation at 8000 *g* for 5 min, suspended in PBS buffer and sonicated. After centrifugation at 25,000 *g* for 40 min at 4°C, the supernatant was loaded onto a Ni–NTA–agarose column followed by removal of the six His-Tag with an enterokinase for 18 h at 37°C. After purification and refolding, the protein was identified by SDS-PAGE and western blotting [33]. The anti-Cry1C polyclonal antibodies and horseradish peroxidase–conjugated goat anti-rabbit IgG (H+L) secondary antibody were synthesized from YouLong (Shanghai, China) and ProteinTech Group (Chicago, USA), respectively.

### Structural and functional equivalence assay

The proteins isolated from rice and *E*. *coli* cells were identified by SDS-PAGE, western blotting and glycoprotein assay. The glycoprotein assay was carried out by using glycoprotein staining (Pierce, Rockford, USA) according to the manufacturer’s instructions.

Cry1C protein derived from *E*. *coli* and rice were thoroughly blended into the diet of first instar larvae of *Chilo suppressalis* to obtain appropriate concentration. Diet without the Cry1C protein was used as control. All bioassays were performed in glass tubes [18, 34]. 3–4 pieces of leaves from TT51 and Minghui 63 were tooken. The veins were sliced, and the middle 6 cm part of the leaves were kept, and then the two ends of which were kept wet with filter papers previously soaked with distilled water. The leaves were then placed in a tube (10 cm length, 1.2 cm diameter). Each tube was inoculated with 12 one-day larvas, jam-packed with cotton ball and kept horizontal. The two ends of the tube about 2 cm long were coverd with black fabric, and were placed in the insectary with room temperature 27±1°C. On the 3rd day, 2–3 pieces of fresh leaves from the same plant were added into the tube. After 6 days, the survival and growth of test larvas were examined, and the survived larves was weighed. The amount of toxin adopted was 21ug/g leaves. TT51 and Minghui 63 were used as positive and negative controls, respectively. TT51 is transgenic rice expressing fusion gene *Cry1Ac/Cry1Ab*, and Minghui 63 is its transgenic receptor. Mortality was calculated a week later.

### In vitro digestibility of the Cry1C protein

*In vitro* digestion of the Cry1C protein was carried out under the guide of Ministry of Agriculture of PR China No. 869 Bulletin 2–2007. The Cry1C protein was added into SGF [105.3 mg of pepsin (1:2500, Sigma, St Louis, Mo, USA), 0.2 g of NaCl, 730 μl of hydrochloric acid, pH 1.2, 100 ml] and SIF [1.0 g of trypsin (1:250, Sigma), 0.7 g of KH_2_PO_4_, pH 7.5, 100 ml], respectively, and the mixtures were incubated at 37°C. The concentrations of the Cry1C protein in SGF and SIF were 0.25 and 0.1 mg/ml, respectively. The ratio of the Cry1C protein (5 and 2 g/l for SGF and SIF, respectively) to enzyme was 1:19. The samples were collected at intervals of 0, 15, 30 and 60 s; and 2, 10, 30 and 60 min; loaded onto SDS-PAGE, and transferred to membranes for western blotting [35]. Ovalbumin (OVA) and BSA were used as positive and negative controls, respectively.

### Allergenicity evaluation of E. coli Cry1C protein in rats

OVA (Sigma, USA), a potent respiratory and food allergen, was determined to have function of serological responses (IgE antibody production) [36]. An ip dosing scheme was used for sensitization.

Five groups of 6–8-week-old female Sprague Dawley rats (n = 6, weight 180–220 g) were used (Vital River Laboratories Co Ltd, Beijing, China). The five groups received 2 ml of aluminum potassium sulfate (90 mg, adjuvant), 10 μg/ml OVA with adjuvant, 250 μg/ml pure *E*. *coli* Cry1C protein and 1000 μg/ml pure *E*. *coli* Cry1C protein in PBS and PBS alone as control, respectively. The rats received 5000mg/kg Cry1C proteins for three times at 0 d. All five groups received the reagents via ip injection at 1, 7 and 14 d. Rats were exsanguinated at day 21st after exposure. Serum sample was prepared. The obtained blood was placed in centrifuge tubes and kept still or placed in 37°C to promote its solidification. Then the coagulated blood was centrifuged at 3000 rpm for 10 min. The obtained supernatant was the required serum, which was then pipetted out and stored at –20°C for use. Protein-specific and total IgE antibodies were detected using ELISA [36].

### Measurement of protein-specific IgG and IgE antibodies

The rats showed no allergic reaction after dermal challenge, therefore the IgG and IgE produced in rats were determined by *in vitro* immunoassay. Protein-specific IgG and IgE antibodies were detected using ELISA, which measured total and specifically induced serum IgG and IgE antibodies for OVA and Cry1C. The horseradish peroxidase–conjugated goat anti-rat IgE and goat anti-rat IgG–HRP antibodies were synthesized by Southern Biotech (Chicago, USA, Cat. Nos. 1110–05 and 1030–05, respectively).

### Acute toxicity test of effect on intestinal bacteria

Rats (weight 180–220 g each) were obtained from the Experimental Animal Center of Peking University (Beijing, China). Nine SPF Sprague Dawley rats (6–7 weeks old, 3 males and 6 females) were used. Following 5 d of acclimatization, they were divided randomly into three groups, with three rats for each group. The concentration of Cry1C was determined at 250 mg/ml to obtain a solution with 95.8% purity. Each rat received 20 ml of protein solution (5 g). To analyze the changes in microbial composition, fresh fecal samples were collected on the day before the feeding study, and then on 1, 7 and 14 d after gavage.

### DGGE analysis of PCR products

Bacterial 16S rDNA was amplified by PCR using the universal primers. DGGE based on the method of Muyzer [37] was carried out using the Dcode^TM^ Universal Mutation Detection System (Bio-Rad, USA). DGGE analysis for V3-16S rDNA products (200 bp) was performed using gels containing a gradient of 30–70% of denaturant. Digital capturing was performed using Diversity Fingerprint Software (Bio-Rad). Quantity One software was used for the detection of bands and normalization of band patterns from DGGE. Cluster analysis was performed based on common and different bands using the binary coefficient Dice. The bands excised from the gels were sequenced by BGI Corporation, and the sequences were analyzed in comparison with the 16S rDNA sequences in GenBank by BLAST search (NCBI) for species identification.

### Statistical analysis

Cluster and diversity analyses based on the DGGE band patterns were performed using NTSYS-pc software package [38]. DGGE gels were analyzed using Quantity One 4.1.1 gel analysis software (Bio-Rad). Bands with intensity < 0.05 were excluded from the analysis. The Shannon–Wiener index (H) of diversity was used to determine the diversity of the bacterial community [39]. The H index increases as the number of species increases or the proportions of the species become more even.

## Ethics Statement

All experiments with the animals were performed in accordance with relevant guidelines and regulations for the Care and Use of Laboratory Animals (Ministry of Science and Technology of China, 2006). All experimental protocols were approved by the animal ethics committee of Shanghai Academy of Agricultural Sciences.

## Supporting Information

S1 File Comment on how the current manuscript advances on previous work. (DOC)

S1 Table Microbial species identification from PCR-DGGE profiles (clones corresponding to the bands marked in Fig 4A, 4F and 4I). (DOC)

S2 Table Cultivation conditions for aerobic and anaerobic bacteria. (DOC)
