# Supplementary material for: Edible Safety Assessment of Genetically Modified Rice T1C-1 for Sprague Dawley Rats through Horizontal Gene Transfer, Allergenicity and Intestinal Microbiota
Source: PLoS One. 2016 Oct 5;11(10):e0163352. doi: 10.1371/journal.pone.0163352 (PMC5051820; doi:10.1371/journal.pone.0163352)
Supplement: S1 File — (DOC) [file pone.0163352.s001.doc]

Comment on how the current manuscript advances on previous work**:**

The method of the study on transgenic rice T1C-1 with the gene *cry1C* is similar to that of others, as the research method in the area is standard, systematic and should be more or less the same, however, a critical safety assessment of transgenic rice T1C-1 was performed for the first time on a case-by-case basis in this study.
