# Supplementary material for: Edible Safety Assessment of Genetically Modified Rice T1C-1 for Sprague Dawley Rats through Horizontal Gene Transfer, Allergenicity and Intestinal Microbiota
Source: PLoS One. 2016 Oct 5;11(10):e0163352. doi: 10.1371/journal.pone.0163352 (PMC5051820; doi:10.1371/journal.pone.0163352)
Supplement: S1 Table — (DOC) [file pone.0163352.s002.doc]

**S1 Table. Microbial species identification from PCR-DGGE profiles (clones corresponding to the bands marked in Figure 4a, f and i).**

| **Band No.** | **Sequence (bp)** | **Closest relatives** | **accession no** | **Identity (%)** |
| --- | --- | --- | --- | --- |
| b1 | 172 | Uncultured bacterium clone T2C113 | [JQ265405.1](http://www.ncbi.nlm.nih.gov/nucleotide/399141433?report=genbank&log$=nucltop&blast_rank=1&RID=7K090B4K014) | 100 |
| b2 | 189 | *Erysipelothrix inopinata* | NR_025594 | 90 |
| b3 | 196 | *Lactobacillus acidophilus* | JX047330 | 100 |
| b4 | 191 | *Bacteroides* sp.TP-5 | AB499846 | 100 |
| b5 | 196 | *Lactobacillus acidophilus* | JX047330 | 100 |
| b6 | 170 | Uncultured Enterobacteriaceae bacterium | JQ683550 | 99 |
| b7 | 174 | Uncultured rumen bacterium | HQ399769 | 99 |
| b8 | 174 | Uncultured *Blautia* sp. | HE974984 | 98 |
| b9 | 196 | *Lactobacillus intestinalis* | JF923643 | 100 |
| b10 | 174 | Uncultured rumen bacterium | HQ400103 | 99 |
| b11 | 173 | *Roseburia intestinalis* | AB661436 | 99 |
| b12 | 169 | Uncultured *Lactobacillus* sp. | AB702903 | 99 |
| b13 | 196 | Uncultured *Lactobacillus* sp. | AB702903 | 99 |
| b14 | 196 | *Lactobacillus acidophilus* | JX047330 | 100 |
| b15 | 190 | Uncultured *Bacteroides* sp. | JN820146 | 99 |
| b16 | 168 | *Clostridiales* bacterium CIEAF 015 | AB702929 | 97 |
| b17 | 172 | *Roseburia intestinalis* | AB661435 | 99 |
| b18 | 189 | *Prevotella* sp. SEQ053 | JN867222 | 97 |
| b19 | 171 | Uncultured Firmicutes bacterium | GU958376 | 99 |
| b20 | 171 | *Roseburia inulinivorans* | AB661436 | 99 |
| b21 | 171 | Uncultured rumen bacterium | GU303277 | 99 |
| b22 | 171 | *Anaerostipes* sp. | JF412658 | 99 |
| b23 | 196 | *Lactobacillus acidophilus* | JX047330 | 100 |
