# Supplementary material for: Edible Safety Assessment of Genetically Modified Rice T1C-1 for Sprague Dawley Rats through Horizontal Gene Transfer, Allergenicity and Intestinal Microbiota
Source: PLoS One. 2016 Oct 5;11(10):e0163352. doi: 10.1371/journal.pone.0163352 (PMC5051820; doi:10.1371/journal.pone.0163352)
Supplement: S2 Table — (DOC) [file pone.0163352.s003.doc]

**S2 Table. Cultivation conditions for aerobic and anaerobic bacteria.**

| Strains to detect | Medium | Aeration | Temperature | Duration |
| --- | --- | --- | --- | --- |
| Anaerobes | MRS culture | nitrogen | 37℃ | 72 h |
| Bacillus coli | Methylthioninium Chloride culture | air | 37℃ | 24 h |
| Bacillus acidi lactici | Improved MC agar culture | air | 37℃ | 48 h |
| Salmonella | SS agar culture | air | 37℃ | 24 h |
| Chain coccus | BMI culture | air | 37℃ | 24 h |
